# Biodiversity of gelatinous macrozooplankton: Quantitative assessment of data and distribution patterns in the southern and central North Sea during August 2018

**DOI:** 10.1016/j.dib.2019.104186

**Published:** 2019-07-09

**Authors:** Christine Gawinski, Bastian Huwer, Peter Munk, Cornelia Jaspers

**Affiliations:** a National Institute of Aquatic Resources, Technical University of Denmark, DTU Aqua, Denmark; b GEOMAR – Helmholtz Centre for Ocean Research Kiel, Evolutionary Marine Ecology, Germany

**Keywords:** Zooplankton, Jellyfish, Comb jelly, Ctenphore, Jellyfication, Global change

## Abstract

This article describes the biodiversity of gelatinous macrozooplankton and presents quantitative field data on their community composition and distribution pattern in the North Sea during August 2018. The data set consists of jellyfish and comb jelly species abundance estimates which are based on sampling at 62 stations in the central and southern North Sea covering Danish waters, the German Bight, waters off the Dutch coast as well as the western North Sea off the UK coast and the central North Sea. The sampling gear was a 13 m long MIK-net (modified Methot Isaac Kidd net; Ø 2 m, mesh size 1 mm, mesh size cod end 500 μm) deployed in double oblique hauls from the surface to 5 m above the sea floor. Samples were visually analysed for gelatinous macrozooplankton (>2 mm) using a light table. Samples were processed within 1 hour after catch. In total, 6239 gelatinous macrozooplankton specimen were caught. Spatial distribution pattern described in this article include the jellyfish species *Aequorea* sp*.*, *Aurelia aurita*, *Beroe* sp., *Chrysaora hysoscella*, *Clytia hemisphaerica*, *Cyanea capillata*, *Cyanea lamarckii*, *Eirene viridula*, *Leuckartiara octona*, *Melicertum octocostatum*, *Obelia* sp*.* as well as the comb jelly species *Mnemiopsis leidyi* and *Pleurobrachia pileus*. Further, size frequency distributions of abundant taxa are provided together with a summary of abundances as well as average, maximum and minimum sizes of all species. This dataset has not previously been published and is of high value for comparison with other – and future - investigations of gelatinous macrozooplankton in the North Sea. The data were obtained during an internationally coordinated, standard fishery survey which is carried out annually (Quarter 3 – North Sea – International Bottom Trawl Survey – Q3 NS-IBTS). The gained information could be used as baseline for a monitoring of potential changes in gelatinous macrozooplankton abundances to address the long standing question if gelatinous zooplankton are on the rise due to climate change induced stressors.

**Table:** Specifications table

Subject area	Biological oceanography, zoology, biodiversity
More specific subject area	Plankton, gelatinous zooplankton, jellyfish, comb jelly
Type of data	5 Figures, 1 Table, 2 Appendices (Appendix 1: specific data description; Appendix 2: link to raw data table).
How data was acquired	Research vessel based plankton survey using a 13 m long MIK-net (modified Methot Isaac Kidd; Ø 2 m, mesh size 1 mm, mesh size cod end 500 μm). Unpreserved samples were analysed on a light table within 1 hour after catch.
Data format	Raw and analysed - including species specific abundances per area (m^−2^) and volume (m^−3^).
Experimental factors	Samples have been analyzed right after catch without preservatives. Data have been standardized to volume specific abundance data by using calibrated flow meter values.
Experimental features	Species identification based on Russell 1953 [1], along with size acquisition of unpreserved material using a caliper on a light table.
Data source location	National Institute of Aquatic Resources, Technical University of Denmark, DTU Aqua, 2800 Kgs. Lyngby, Denmark.
Data accessibility	Data are provided within this article.

**Table:** 

**Value of the data** • This dataset is important for assessing the biodiversity of gelatinous macrozooplankton in the North Sea (>2 mm) and for addressing the long standing scientific questions i) if gelatinous zooplankton are on a rise due to climate change induced stressors (e.g. warming, acidification) and ii) if we observe an increase in non-indigenous macrozooplankton species in the North Sea due to global change (e.g. increased shipping activity, over-fishing). • The applied methodology to obtain this dataset could be used as model for including gelatinous macrozooplankton analyses into existing, standard fishery surveys. • The here presented data could be used as a baseline for future monitoring initiatives of gelatinous macrozooplankton in the North Sea. • Considering increased pressures marine ecosystems are facing due to global change, the data are of high relevance in relation to the European Commission's Marine Strategy Framework Directive (MSFD) and could foster collaboration with European partners which are obliged to assess and achieve good environmental status of marine habitats in their national waters.

## 1 Data

**Fig. 1:**
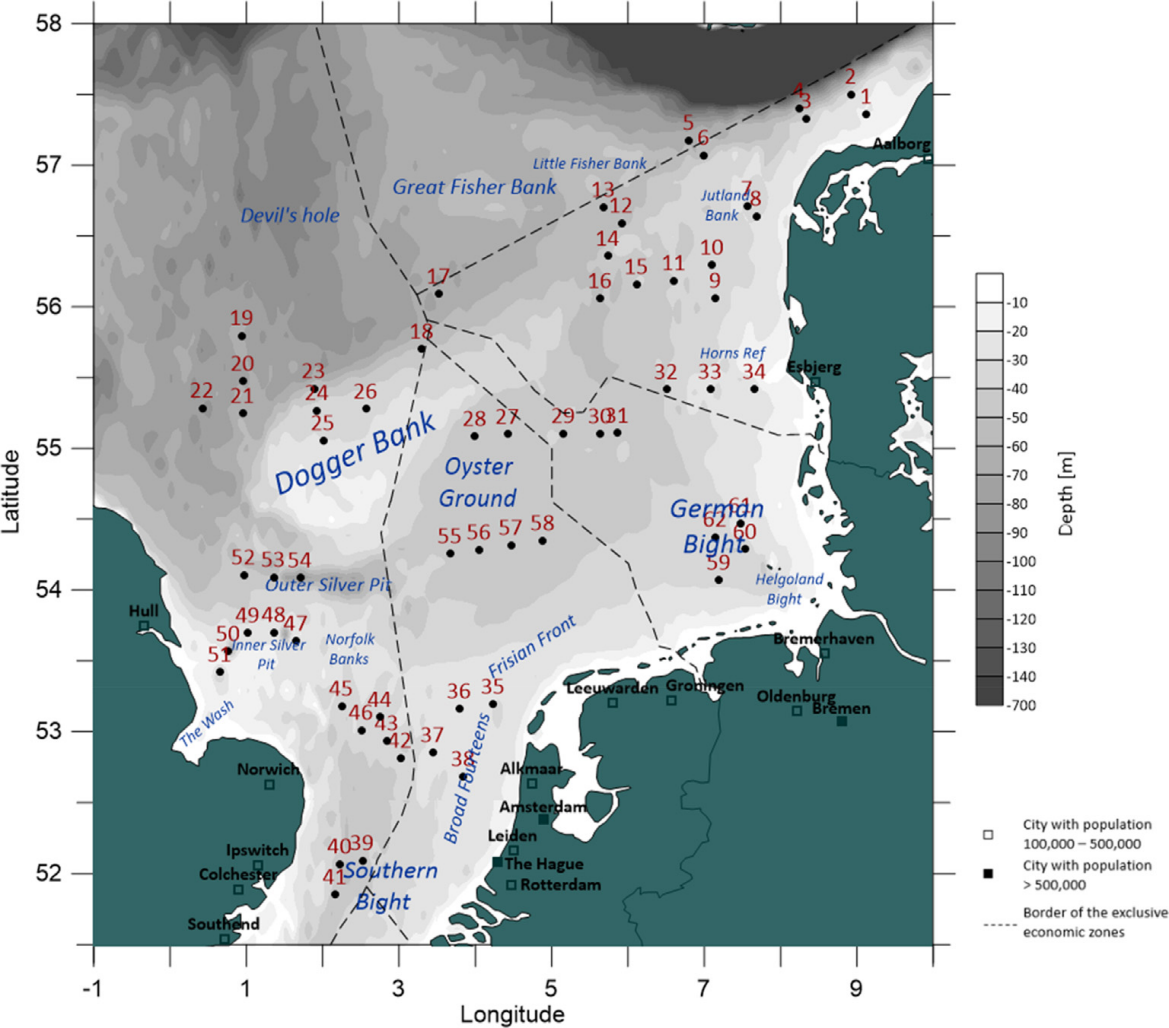
Investigation area of the North Sea where gelatinous macrozooplakton has been sampled at 62 stations (indicated by red number) during August 2018. Basin names are provide (in blue) along with borders of exclusive economic zones - modified from Copejans & Smiths 2011.

**Fig. 2:**
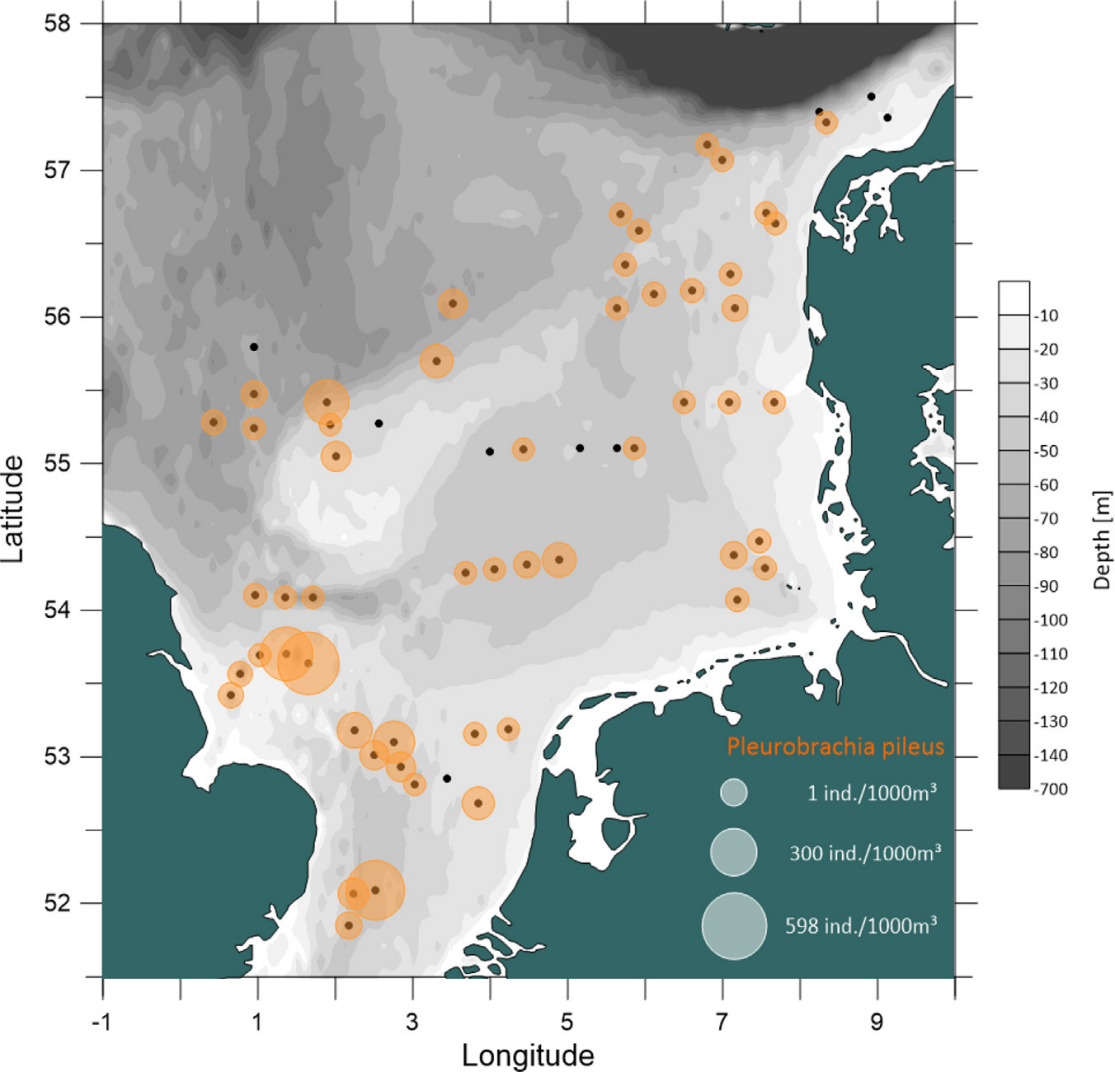
Spatial distribution of the comb jelly *Pleurobrachia pileus* in the North Sea in August 2018. Black dots indicate sampling stations where no animals have been caught.

**Fig. 3:**
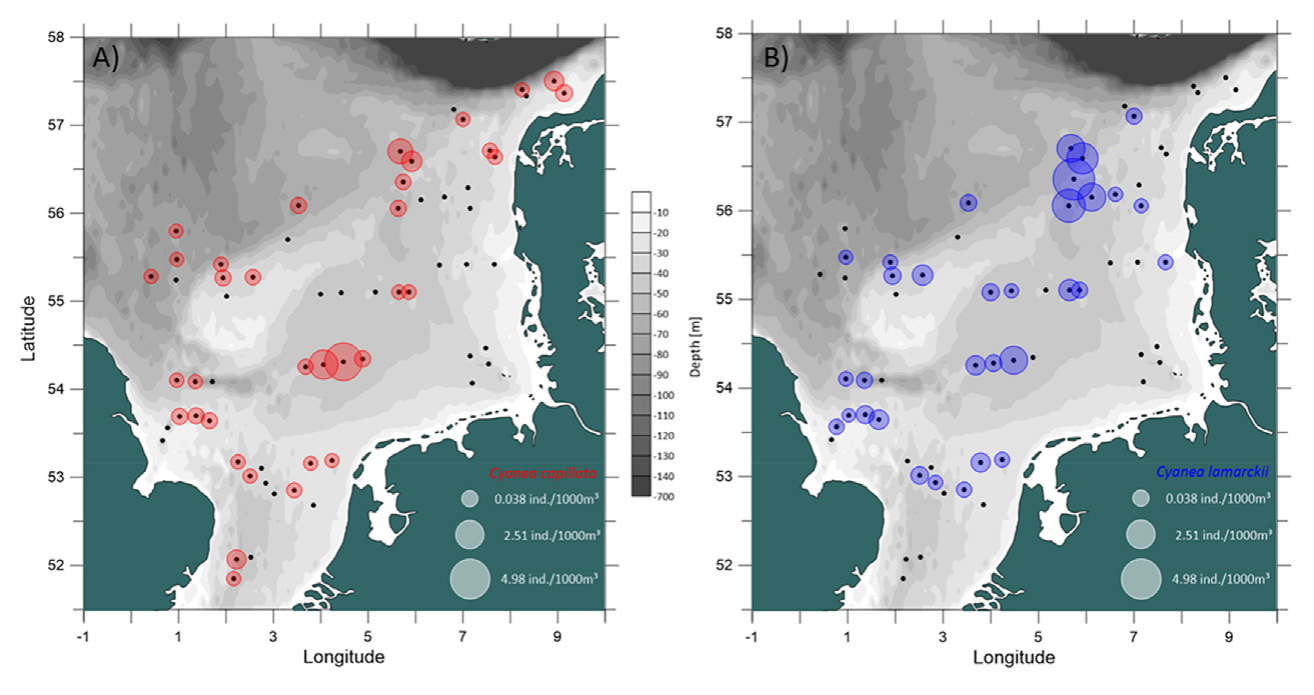
Spatial distribution of the scyphozoan jellyfish species *Cyanea capillata* (A) and *C. lamarckii* (B) in the North Sea in August 2018. Black dots indicate sampling stations where no animals have been caught.

**Fig. 4:**
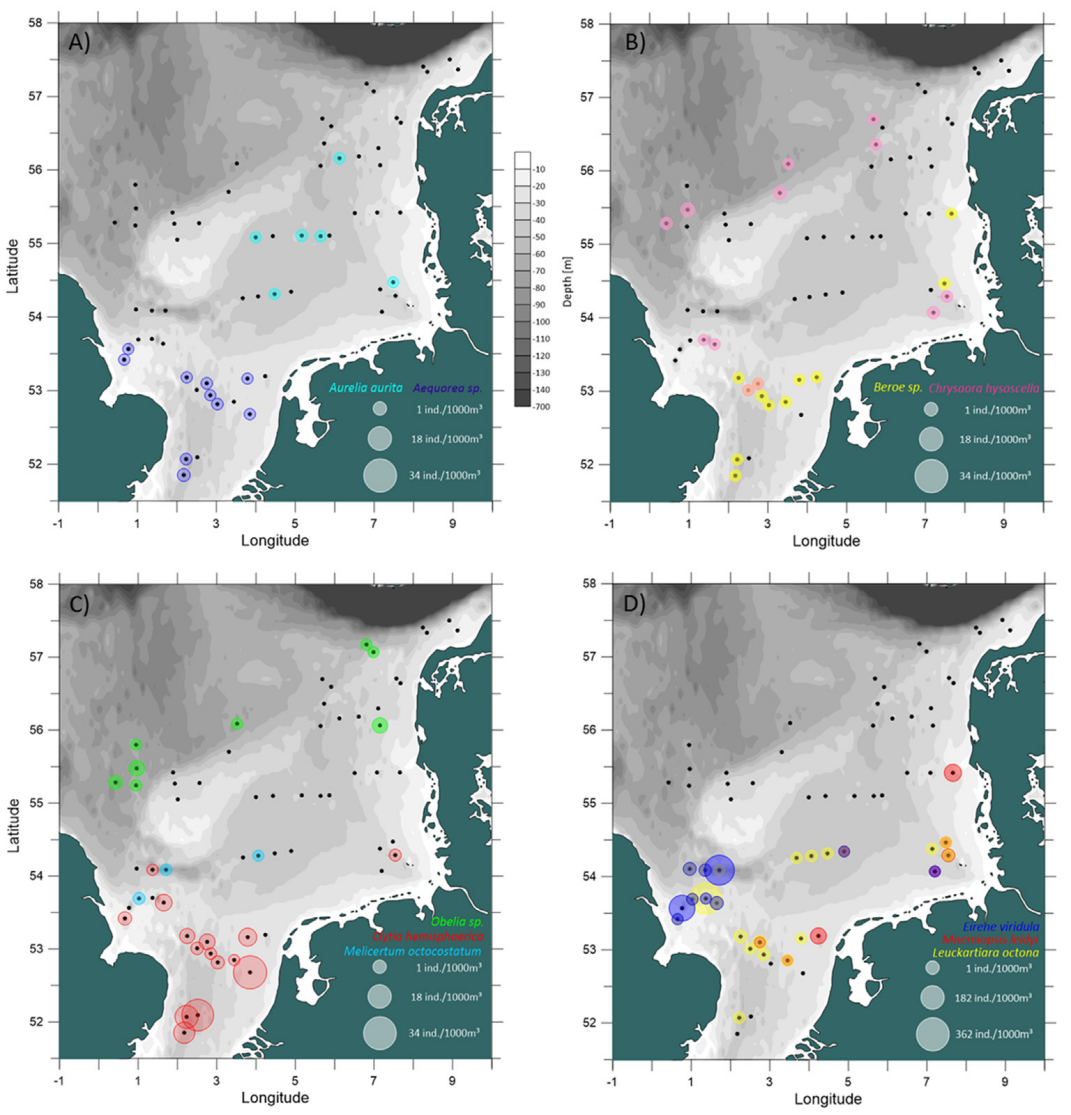
Spatial distribution of gelatinous macrozooplankton species in the North Sea in August 2018 with A) *Aurelia aurita* (light blue) and *Aequorea* sp. (dark blue); B) *Beroe* sp. (yellow) and *Chrysaora hysoscella* (pink); C) *Obelia* sp. (green), *Clytia hemisphaerica* (red) and *Melicertum octocostatum* (lightblue); and D) *Eirene viridula* (blue), *Mnemiopsis leidyi* (red) and *Leuckartiara octona* (yellow). Note: A, B, C use the same scale for depicting abundance data, while D uses a different scale accounting for 1 order of magnitude higher abundance data. Black dots indicate sampling stations where no animals have been caught.

**Fig. 5:**
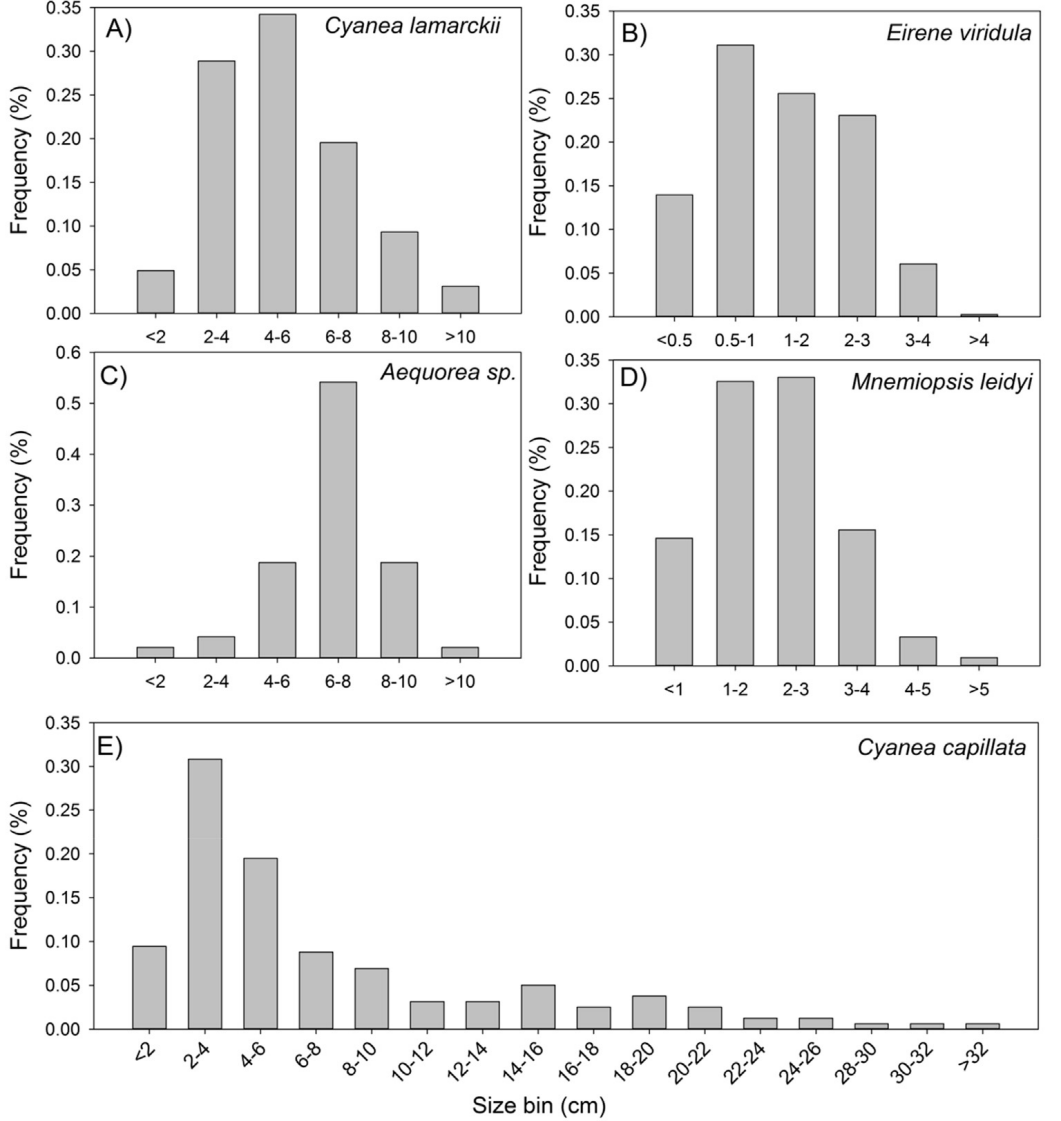
Relative size frequency distribution of selected gelatinous macrozooplankton species in the North Sea during August 2018. Size bins for 1 or 2 cm size classes depicted for the species *Cyanea lamarckii* (A), *Eirene viridula* (B), *Aequorea* sp. (C), *Mnemiopsis leidyi* (D) and *Cyanea capillata* (E).

This data article presents a description of the biodiversity of the gelatinous macrozooplankton community sampled at 62 stations across the southern and central North Sea (Fig. 1) during August 2018. The data consist of spatial distribution patterns and abundance data (Fig. 2, Fig. 3, Fig. 4) of 13 major gelatinous macrozooplankton species along with size frequency distributions of the five most important species (Fig. 5). Further, a table presenting total numbers, average and maximum abundance data across stations as well as average, maximum and minimum sizes is provided (Table 1). A total of 6239 jellyfish and comb jelly specimen were caught belonging to the following species: *Aequorea* sp*.*, *Aurelia aurita*, *Beroe* sp*.*, *Chrysaora hysoscella*, *Clytia hemisphaerica*, *Cyanea capillata*, *Cyanea lamarckii*, *Eirene viridula*, *Leukartiara octona*, *Melicertum octocostatum*, *Mnemiopsis leidyi*, *Obelia* sp*.* and *Pleurobrachia pileus* (see Appendix 1 for detail). All raw data are included in Appendix 2.

**Table 1:** Total counts (N), average (av.) and maximum (max.) abundance as well as average, minimum (min.) and maximum size of gelatinous zooplankton caught in the North Sea during August 2018.

Species	N	Av. abund. 1000m^−^³ ± SD	Max. abund. 1000m^−^³ ± SD	Av. size (cm) ± SD	Min. size (cm)	Max. size (cm)
*Aequorea* spp*.*	48	1.0 ± 1.1	3.7	7.4 ± 1.7	2	12
*Aurelia aurita*	20	0.8 ± 0.8	2.2	10.7 ± 5.9	4	26
*Beroe* spp*.*	78	2.3 ± 2.8	8.9	2.1 ± 1.1	1	5
*Chrysaora hysoscella*	22	0.4 ± 0.3	1	5.5 ± 2.2	2	11
*Clytia hemisphaerica*	186	10.9 ± 12.2	40.5	0.3 ± 0.1	0.3	1
*Cyanea capillata*	159	0.7 ± 0.9	5	8.2 ± 6.7	1	32
*Cyanea lamarckii*	225	1.3 ± 1.4	5.7	5.7 ± 2.2	1	12
*Eirene viridula*	759	54.7 ± 107.2	341	1.8 ± 1.1	0.5	5
*Leuckartiara octona*	442	26.9 ± 70.4	362.7	0.4 ± 0.2	0.3	1
*Melicertum octocostatum*	15	2.5 ± 1.5	4.6	0.3 ± 0.1	0.3	0.5
*Mnemiopsis leidyi*	424	23.8 ± 40.9	109.7	2.6 ± 1.1	1	6
*Obelia* spp.	85	4.4 ± 7.6	26.8	1.8 ± 1.1	0.5	5
*Pleurobrachia pileus*	3776	70.7 ± 128.7	598.7	0.4 ± 0.3	0.3	2
total	6239					

## 2 Experimental design, materials, and methods

Samples were collected in the central and southern North Sea (Fig. 1) at night time (19:30–5:30 GMT) in August 2018 (30.7.2018–16.8.2018) during the Danish contribution to the International Bottom Trawl Survey (IBTS) on board the Danish R/V DANA (DTU Aqua, Denmark). The IBTS is a long-term fishery monitoring program [2] which is conducted twice a year, both in the 1st and 3rd quarter. The standard procedure during the survey is bottom trawling during daytime to provide abundance indices for a range of commercially important fish species, as well as standard CTD casts to describe the physical parameters. During the Danish 3rd quarter IBTS in 2018 these standard procedures were supplemented by plankton sampling during nighttime.

During this additional plankton sampling, gelatinous macrozooplankton was sampled on a total of 62 stations across the southern and central North Sea (Fig. 1) by use of a MIK-net (modified Methot Isaac Kidd) net. The MIK-net is a large ring net with a 2 m diameter mouth opening and a 13-m-long net with a mesh size of 1 mm. The last metre of the net as well as the cod end bucket have a finer mesh size of 500 μm. The net was hauled at a speed of 3 knots in a double oblique tow from the surface to 5 m above the sea floor. A calibrated flow meter in the center of the gear opening was used to assess the water volume filtered during the tow. A total of 69 hauls were analysed for gelatinous macrozooplankton, with duplicated hauls at stations 9, 12, 38, 42 and 55 and triplicated hauls at station 59. After the net had been retrieved and carefully washed, the un-preserved cod end contents were sorted for gelatinous macrozooplankton (>2 mm) and fish larvae on a light table in R/V DANA's laboratory. All jellyfish and comb jellyfish were identified to genus or species level [1]. Jellyfish and comb jellyfish were rinsed and individually removed from the sample, whereafter they were counted and sized to the nearest mm using a caliper. The remaining zooplankton sample was concentrated on a sieve (mesh size 150 μm) and preserved in 96% ethanol within 1 h after catch.

The amount of filtered water in m³ per station was calculated using the following formula:

$$Filtered\;water\;per\;station\;\left[m^{3}\right]=\;\frac{\Delta \;flowmeter\;count}{34.96}\;*\;\pi \;*\;radius²$$

whereΔ flowmeter count = the difference of the flowmeter values before and after the haul, 34.96 = a flowmeter calibration coefficient determined during calibration hauls and radius = 1 m. Total counts of organisms per station were divided by the amount of filtered water per station to calculate individuals per m³. In order to avoid very small values, these abundance data were further standardized to 1000 m^3^. Average abundance data along with size information are provided in Table 1.

The spatial distribution of gelatinous macrozooplankton were visualized using the Software Surfer^®^ (Golden Software LLC). Bathymetry data for surfer maps were obtained as ESRI ASCII files from the EMODnet Data Portal (http://portal.emodnet-bathymetry.eu/) for tiles D3, D4, E3 and E4, using the DTM version 2018. Sampling stations were plotted according to their coordinates and associated gelatinous macrozooplankton abundance data were depicted as circles with the size of the circle proportional to the abundances (Fig. 2, Fig. 3, Fig. 4). Size frequency distributions were calculated for the most important five species only (Fig. 5).

Hydrography: Physical parameters from the North Sea during the investigation period can be downloaded from the ICES hydrographic database.

## Appendix 1 Specific data summary

***Pleurobrachia pileus:*** A total of 3776 individuals were caught at 53 stations. These ctenophores were prevalent along the Inner Silver Pit, with a maximum abundance of 599 individuals 1000 m^-^³ at station 47 (Table 1). Overall, their average abundance was 71 individuals 1000 m^-^³ and they were found at all stations except station 1, 2, 4, 19, 26, 28, 29, 30 and 37 (see Fig. 2). The mean size was 0.43 cm ± 0.33 cm (Table 1) and the sizes ranged between 0.25 to 2 cm (see Fig. 5).

***Cyanea capillata:*** A total of 159 individuals were found at 35 stations throughout the investigated part of the North Sea except for the German Bight (see Fig. 3A.). The scyphozoan jellyfish had a mean abundance of 0.7 individuals 1000 m^-^³ and were most abundant on station 57 with 5 individuals 1000 m^-^³. The mean size was 8.17 ± 6.67 cm with a size range between 1 to 32 cm (see Fig. 5).

***Cyanea lamarckii:*** This scyphozoan jellyfish was most abundant along the Great Fisher Bank as well as the Little Fisher Bank (see Fig. 3B). A total of 225 medusae were found at 32 stations, with a mean and maximum abundance of 1.3 and 5.7 individuals 1000 m^-^³, respectively. The size of *Cyanea lamarckii* varied between 1 to 12 cm with a mean size of 5.7 ± 2.2 cm (see Fig. 5).

***Aurelia aurita:*** This scyphozoan jellyfish was caught at 6 stations in the German Bight and south of the Jutland Bank (Fig. 1, Fig. 3A). Mean and maximum abundances were 0.8 and 2.2 individuals 1000 m^-^³, respectively. The size of *A. aurita* ranged between 4 to 26 cm with a mean size of 10.7 ± 5.9 cm (Table 1).

***Aequorea* sp.:** Were found at 10 stations, being most abundant in the Southern Bight of the North Sea, while they were found in low numbers along the West Coast of Great Britain, near The Wash (Fig. 4A). A total of 48 individuals were caught with a mean and maximum abundance of 1 and 3.7 individuals 1000 m^-^³, respectively (Table 1). The average size was 7.4 ± 1.7 cm (see Table 1, Fig. 5).

***Beroe* sp.:** Were found along the Great Fisher Bank, the Little Fisher Bank, north-west and north-east of the Dogger Bank, north of the Inner Silver Pit, south of the Norfolk Banks and in the western Helgoland Bight (see Fig. 3). At 12 stations 78 individuals were caught. The mean and maximum abundance was 2.3 and 8.9 individuals 1000 m^-^³, respectively. The sizes ranged between 1 to 5 cm with a mean size of 2.1 ± 1.1 cm (Table 1).

***Chrysaora hysoscella*:** Most of these jellyfish were caught in the Southern Bight and along the Broad Fourteens, some individuals were occasionally encountered in the German Bight and North of the North Frisian Islands (see Fig. 4). The mean and maximum abundance was 0.4 and 1 individual 1000m^-^³, respectively. The mean size was 5.5 ± 2.2, with a size range of 2 to 11 cm (Table 1).

***Obelia* sp.:** A total of 85 *Obelia* sp. were caught on 8 stations (Fig. 4C). The jellyfish were most abundant north-west of the Dogger Bank and on the west-coast of Denmark, they were occasionally encountered north of the Jutland Bank and in the North of the Dogger Bank (see Fig. 4C). The mean and maximum abundance was 4.4 and 26.8 individuals 1000 m^-^³, respectively (Table 1). The size ranged between 0.5 to 5 cm with an average size of 1.8 ± 1.1 cm (Table 1).

***Clytia hemisphaerica*:** A total of 186 individuals were caught at 15 stations. They were most abundant in the Southern Bight and along the Broad Fourteens, with a mean and maximum abundance of 10.9 and 40.5 individuals 1000 m^-^³, respectively (Fig. 4C). The size ranged between 0.25 to 1 cm, with an average size of 0.7 ± 0.1 cm (Table 1).

***Melicertum octocostatum:*** Were caught at 3 stations in the Inner Silver Pit, close to the coast of Great Britain and occasionally encountered in the open waters of the Oyster Ground (Fig. 4C). Their mean and maximum abundance was 2.5 and 4.6 individuals 1000 m^-^³, respectively (Fig. 4C). The size ranged between 0.25 to 0.5 cm and the mean size was 0.30 ± 0.10 cm (Table 1).

***Eirene viridula:*** *Eirene viridula* was one of the most abundant species with a mean and maximum abundance of 54.7 and 341 individuals 1000 m^-^³, respectively. A total of 759 individuals were caught at 10 stations. The jellyfish were found in highest numbers west of the Dogger Bank, namely along the Outer Silver Pit, on the Norfolk Banks and Inner Silver Pit (see Fig. 4D). Occasionally they were encountered in the German Bight. The size ranged between 0.5 to 5 cm with an average size of 1.84 ± 1.05 cm (Table 1, Fig. 5).

***Mnemiopsis leidyi:*** The comb jelly *Mnemiopsis leidyi* was found south of the Norfolk Banks, along the Broad Fourteens, the Frisian Front and in the German Bight (see Fig. 4D). A total of 424 individuals were found at 8 stations, with an average and maximum abundance of 23.8 and 110 individuals 1000 m^-^³, respectively (Fig. 4D). The mean oral-aboral size of *Mnemiopsis leidyi* was 2.6 ± 1.1 cm (Fig. 5).

***Leuckartiara octona:*** Was the second most abundant gelatinous macrozooplankton species with a mean and maximum abundance of 26.9 and 362.7 individuals 1000 m^-^³, respectively. The jellyfish were found at 21 stations in the southern and central parts of the North Sea, namely the Southern Bight, south-west of the Dogger Bank and in the German Bight (Fig. 4D). A total of 442 individuals with a size range between 0.25 to 1 cm were caught with an average size of 0.4 ± 0.2 cm (Table 1).
